# Molecular Classification of Gastrointestinal and Pancreatic Neuroendocrine Neoplasms: Are We Ready for That?

**DOI:** 10.1007/s12022-024-09807-2

**Published:** 2024-03-12

**Authors:** Silvia Uccella

**Affiliations:** 1 Department of Biomedical Sciences, Humanitas University, Via Rita Levi Montalcini 4, 20072 Pieve Emanuele, Milan, Italy; 2 Pathology Service IRCCS Humanitas Research Hospital, Via Manzoni 56, 20089 Rozzano, Milan Italy

**Keywords:** Neuroendocrine neoplasm, Neuroendocrine tumor, Neuroendocrine carcinoma, Molecular classification, Target therapy, Genetics

## Abstract

In the last two decades, the increasing availability of technologies for molecular analyses has allowed an insight in the genomic alterations of neuroendocrine neoplasms (NEN) of the gastrointestinal tract and pancreas. This knowledge has confirmed, supported, and informed the pathological classification of NEN, clarifying the differences between neuroendocrine carcinomas (NEC) and neuroendocrine tumors (NET) and helping to define the G3 NET category. At the same time, the identification genomic alterations, in terms of gene mutation, structural abnormalities, and epigenetic changes differentially involved in the pathogenesis of NEC and NET has identified potential molecular targets for precision therapy. This review critically recapitulates the available molecular features of digestive NEC and NET, highlighting their correlates with pathological aspects and clinical characteristics of these neoplasms and revising their role as predictive biomarkers for targeted therapy. In this context, the feasibility and applicability of a molecular classification of gastrointestinal and pancreatic NEN will be explored.

## Introduction

The definition and classification of neuroendocrine neoplasms (NEN) have been subject to ongoing refinement for the last years. On one hand, the distinction between neuroendocrine tumor (NET) and neuroendocrine carcinoma (NEC), supported by histopathological features, molecular hallmarks, and clinical evidence, has provided a solid base for therapeutic management [1, 2]. On the other hand, the “NEN concept” has been discussed for neoplastic proliferations arising outside the “classic” thoracic and digestive sites, with different outcomes. For instance, the nomenclature shift from *pituitary adenoma* to *pituitary neuroendocrine tumor-PitNET* has acknowledged the morpho-functional properties of these neoplasms and has also taken in account their clinical correlates, in terms of both endocrine function and oncological manifestations [3, 4]. In contrast, for breast cancers, the application of the “NEN concept” has not proven to be completely justified by well-defined morphological and biological characteristics and, as a matter of fact, is not useful in terms of patient management, at least at the present moment [5–7]. In general, the definition and the classification of NENs are currently based on well-established morphological criteria, supported by the expression of specific general neuroendocrine markers (synaptophysin, chromogranin A, and INSM1), and related with predictable clinical behavior and response to therapy [8]. These concepts have been clarified in the latest edition of the WHO classification of neuroendocrine neoplasms and have been extended to extra-thoracic and extra-digestive primary sites [8]. The application of this classification framework in real-life series is needed to validate this approach in so-called “unusual sites” of NENs, namely, the urinary tract, the female and male genital systems, and the head and neck region.

The cornerstone of the current classification scheme of NEN in every primary anatomical location is the identification of the morphological degree of differentiation, that acknowledges the existence of well-differentiated NEN (NET) versus poorly differentiated NEN (NEC) and is most relevantly related to clinical behavior [8, 9]. Importantly, a great burden of molecular data has accumulated supporting two separated pathogenetic mechanisms for NET and NEC, with the involvement of distinct intracellular an microenvironmental pathways [10–12]. This knowledge has two main consequences: first, distinct molecular targets may be identified for the tailored treatment of NET and NEC [2]; second, the progression or transformation of a NET to a NEC is an unlikely event [13–15]. Regarding this latter statement, it is worth noting that in the lung, possibly related to the smoke-related context, a subset of NEC has been demonstrated to be molecularly related to pre-existing NET, challenging the apodictic paradigm of double-edged NEN [16–18]. In both the NET and NEC families, a biological and clinical heterogeneity is acknowledged, necessitating the identification of more and more specific histopathological criteria and biomarkers to properly address the complex therapeutic management of patients with NEs [19].

In the field of NET, the role of Ki67 proliferation index (PI) in stratifying the risk of individual patients has been validated, and a three-tiered grading system based on specific cut offs of KI67 PI has been introduced for digestive NET [20]. In fact, the prognostic role of KI67 PI is well-established in pancreatic NET, whereas it is less defined in NET of other primary anatomical sites, even inside the digestive system, i.e., the small bowel, or for specific types of tumors, e.g., ECL-cell NET of the stomach [21]. For extra-digestive NET, including pulmonary ones, the prognostic role of Ki67 is still matter of debate, despite its undoubted usefulness in specific diagnostic contexts and for general enframement of these neoplasms [21, 22]. Importantly, about ten years ago, it became evident that high values of Ki67 PI (i.e., > 20%) were not specifically associated with digestive NEN showing poorly differentiated morphology and clinicopathological features of NEC and that so-called high grade NEN were not a monolithic entity [23–25]. These observations led to the recognition of morphologically well differentiated NENs with Ki67 PI higher than 20%, that were named grade 3 NET (G3 NET) and were described in the pancreas and in other digestive organs [19, 20, 26]. Similarly, in the lung, NET with elevated mitotic counts (> 10 mitosis/2 mm^2^) and/or Ki-67 PI (> 30%) were described [27, 28]. In the light of molecular studies, these highly proliferating NET are currently interpreted as the pulmonary counterpart of digestive G3 NET [10, 15, 29]. As G3 NET are relatively rare, the standardization of therapeutic strategies for this category of NEN is yet to be optimized, as well as their pathogenetic mechanisms are still to be defined. However, it seems to be evident that these NENs should be considered separately from both low-grade NET and NEC [30, 31]. Despite the great importance of Ki67 PI values, in daily clinical practice, they are not able, at least using the currently employed cut-offs, to explain the variability of clinical behavior and response to therapy of NET and other biomarkers are needed to stratify patients’ risk, to guide treatment choices and to provide new target for effective drugs [19, 21].

Regarding NEC, until recently, these neoplasms have been considered so furiously aggressive and with such a universally ominous prognosis that they were not worth of being further defined and classified. Nevertheless, new knowledge has been accumulating in the last years regarding different pathogenetic mechanisms underlying not only the morphological and clinical differences between small cell and large cell subtypes of NEC but also the spectrum of diversities observed inside the two subtypes [10]. Clear-cut morphological criteria for the distinction between the large cell and small cell subtypes have been defined, as well as for the differential diagnosis with potential mimickers [8]. In addition, the molecular relationships of NEC, mostly of the large cell subtype, and the non-neuroendocrine carcinomas arising in the various primary sites have been explored, also in the context of mixed neuroendocrine/non-neuroendocrine neoplasms (MiNEN), providing important information on the development and progression of these NENs [10].

In this complex scenario, in which the definition and classification of NEs is a continuously ongoing process, this review article will address the current knowledge on the molecular features of digestive NEN and their relationships with the current classification framework. On these bases, we will also explore the feasibility of a molecular classification of these neoplasms.

## The Molecular Landscape of Digestive NEN

The molecular and genetic landscape of digestive NENs is heterogeneous and varies according to the degree of differentiation, the site, and, regarding NET, proliferation grade. Due to the rarity and diversity of these neoplasms, their comprehensive molecular profiling has been addressed only in a few studies, from which, however, several important conclusions may be driven. Before going into the details of each group of digestive NENs, some general statements may be established, as follows.

- ***NEC and NET have different molecular alterations.*** There is a consolidated burden of evidence that the morphological, biological, and clinical differences between NEC and NET underlies important genomic differences. This is related not only to the alteration of specific genes involved in the pathogenesis but also to genome-wide differences, including tumor mutational burden (TMB), the number of single-nucleotide variants (SNV) and multiple nucleotide variants (MNV), microsatellite instability (MSI), and ploidy and copy number variations (CNV) [32–36]. Moreover, epigenetic alterations are also differentially involved in NET and NEC [37]. In general, NEC of the digestive sites and NEC of other anatomical sites are most frequently characterized by mutations of *TP53* and *RB1*, together with other key driver genes, including, but not limited to, *RAS* family, *APC*, *CDKN2A*, and *MYC* [13, 14, 32–34, 36, 37]. In addition, NEC display high genome instability, with diploid to triploid genome, a median TMB comparable to that of non-neuroendocrine aggressive carcinomas, high numbers of SNV and MNV, and frequent structural chromosomal alterations, including, in a subset of cases, the catastrophic event of chromothripsis [32, 33, 36]. Related to this scenario, NEC may display single- or double-strand DNA-repair deficiency and high tumor neoantigen burden, which are importantly involved in the increased immunogenicity of the neoplasm [36]. In contrast, the molecular landscape of gastroenteropancreatic NET (similarly to their pulmonary counterpart) lacks mutations of key cancer genes and is characterized by genetic and epigenetic alterations of genetic pathways related to chromatin remodeling and telomeres maintenance (e.g., *MEN1*, *ATRX, DAXX*, and *ARID1A* genes), PI3K/Akt/mTOR signaling (e.g., *PTEN* and *TSC2* genes), and VEGF pathway, and cell cycle regulation (e.g., *CDKN1A* and *CDKN1B* genes) [14, 32, 33, 36, 38]. In addition, a non-negligible proportion of NET (up to 10%) arises in the context of an inherited tumor syndrome, determined by germline mutations of specific genes (e.g., *MEN1*, *CDKN1B*, *VHL*, *NF1*, *TSC1/2*, *PTEN*, *GCGR*, and *MAFA* genes) that are also been proven to be involved in the pathogenesis of sporadic digestive NET [14, 39]. At a genome-wide level, NET show a flat diploid genome, a very low median TMB, and low numbers of SNV and MNV [32]. As for structural chromosomal alterations and CNV, NET seem to differ according to the primary site of insurgence of the neoplasm [32, 33]. Due to their stable genome, NET (at least for G1-G2 NET) display a low TNB, overall being non-immunogenic neoplasms [36]. In turn, epigenetic changes seem to play an important role in driving the pathogenesis of NET of all sites, inducing molecular alterations of cancer-related genes and activation of carcinogenetic processes that may also modulate the function of important tumor suppressor genes like *TP53* and *RB1* that are not typically mutated in NET [11, 40].
- ***Grade 3 (G3) NET have similar molecular landscape to G1-G2 NET; however, a subset of them may show some overlapping features with NEC. ***As shown in the previous paragraphs, the dichotomic approach to the classification of NENs, i.e., the distinction between NET and NEC, has solid biological and clinical bases and has contributed to the clarification of the diagnostic and therapeutic management of patients [41]. However, the recently recognized category of G3 NET, although well-defined histopathological criteria for its diagnosis have been established, still poses some problematic issues regarding the definition of its molecular features and, consequently, of the patients’ management. G3 NET are relatively rare (less than 10% of digestive NENs), are frequently of pancreatic origin, and are commonly diagnosed at metastatic sites [42]. This latter feature, as well as their frequent association with G1-G2 NET components in the primary site [43], suggests that G3 NET may represent a phenomenon of progression of lower grade NET, due to a stepwise acquisition of additional molecular alterations. This is related to the general concept of spatial and temporal heterogeneity of NET that has recently been considered for its important practical implications on patients’ management [44]. In fact, little is known about the specific molecular profiles of G3 NET, except for the pancreatic site, where it has been demonstrated that *TP53* mutations are not exclusive of NEC and may be also observed in G3 NET [45]. In pancreatic G3 NET, however, *TP53* mutations are present in the typical genomic background of NET and are neither coupled with other key cancer genes mutations, such as *RB1* or *CDKN2A*, nor with high TMB or other signs of genomic instability [45]. Thus, it can be hypothesized that, in G3 NET, *TP53* mutations may not be related to the early steps of carcinogenesis and may rather be interpreted as a later step in tumor progression. However, scientific evidence about the molecular profiles of G3 NET is still too scant to drive definitive conclusions and further studies are needed to elucidate this issue.
- ***Both NEC and NET of the digestive tract display distinct molecular alterations in different primary anatomical sites.*** There is increasing evidence supporting the view that the specific site of insurgence of a NEN, whether a NET or a NEC, is related to distinct pathogenetic events, possibly related to different local microenvironment and acting risk factors. This is detectable at a molecular level, with the involvement of specific genomic alterations [10, 14, 32, 37, 46], but also non-genomic mechanisms have been invoked, including, but not limited to, the composition of local microbiota [47], the interactions with stromal and inflammatory cells [48], and the action of specific local growth factors and growth factors receptors [49]. Details will be addressed in the paragraphs dedicated to each type of NEN.

## Molecular Features of Digestive NEC

NEC of the various anatomical locations of the digestive tract share the genomic features mentioned in the previous paragraph, in terms of genome-wide alterations (TMB, SNV, MNV, and CNV) and key cancer genes involved. However, site-dependent variability of the molecular landscape of these neoplasms has been reported [10, 35, 46, 50]. Moreover, recent lines of investigation have explored the possibility of molecular subtyping of digestive NEC with the same criteria used for high-grade neuroendocrine neoplasms of the lung [51, 52]. The recognition of the heterogeneous molecular background of digestive NEC has important potential implications for patients’ management. The identification of specific druggable molecular targets may overcome the “one size fits all” concept that drives the administration of platinum-based chemotherapeutic schedules to all patients with NEC and lead to more personalized and effective therapies.

### Site-Specific Molecular Alterations in Digestive NEC

Due to relative the rarity of digestive NEC, compared to their pulmonary counterpart, the systematic comparative analysis of their molecular alterations has been poorly addressed in the past. In the second and third decades of this century, studies investigating the molecular features of NEC of single digestive sites (e.g., pancreas, colon, and stomach) were performed [14, 53–62]. Overall, these studies contributed to demonstrate that NET and NEC of the same sites were genetically different. On the other hand, they also showed that NEC of different sites shared many similarities with adenocarcinomas arising in the same organ. This latter acquisition was also supported by the existence, in these sites, of mixed neuroendocrine/non-neuroendocrine neoplasms (MiNEN), in which the two components show common driver mutations, that are also shared by pure NEC [63]. The conceptual integration of the data deriving from these studies brought to the deduction that NEC of different sites had distinct molecular profiles, in which key cancer genes were differentially involved [10].

**Fig. 1:**
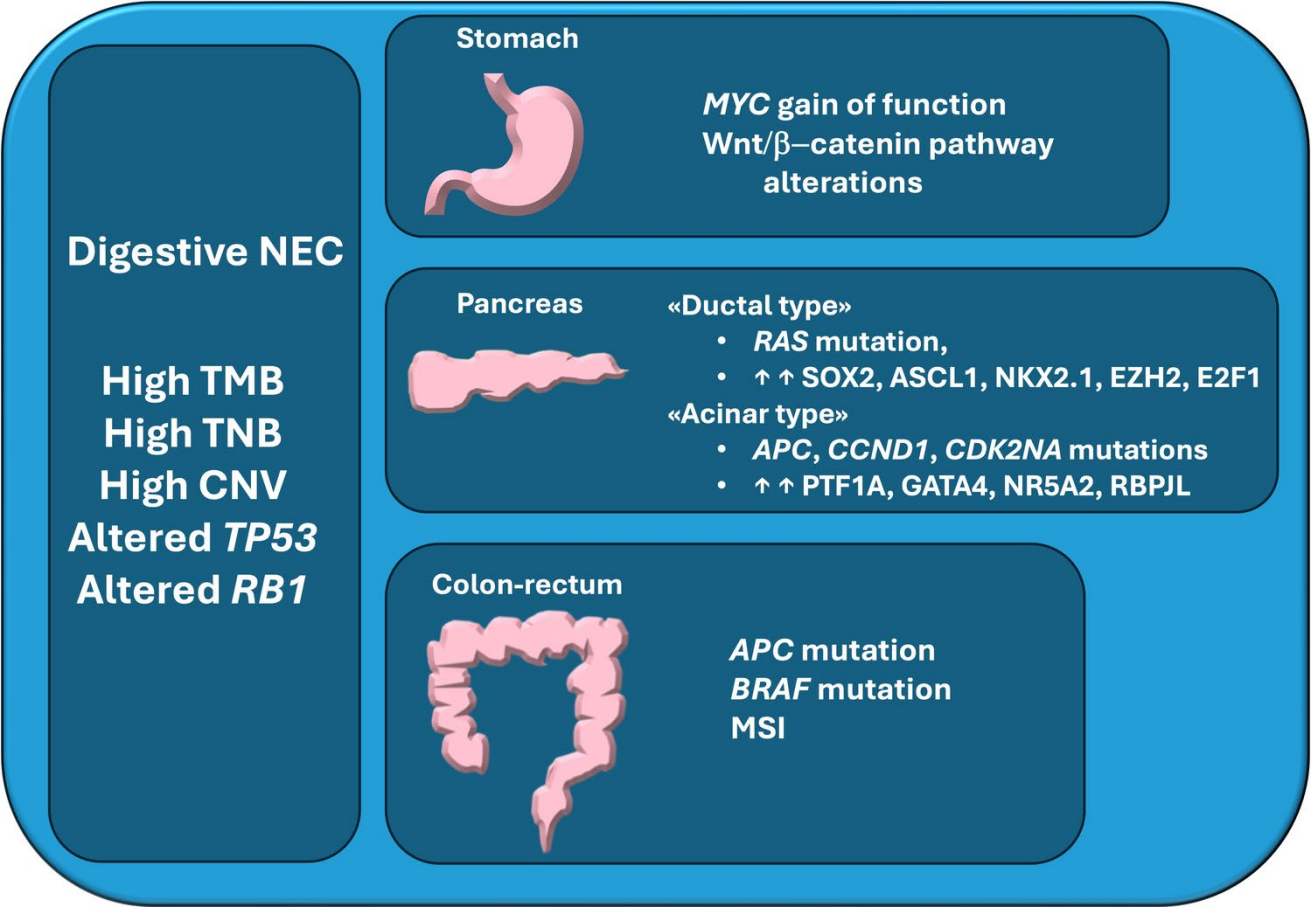
Summary of site-specific molecular alterations in digestive NEC. TMB, tumor mutation burden; TNB, tumor neoantigen burden; CNV, copy number variation; ↑↑ increased expression; MSI, microsatellite instability

Recently, the concept of a site-specific heterogeneity of NEC was addressed using comprehensive approaches, with the analysis of large series of neoplasms from different anatomical location in the digestive tract through genome-wide technologies [35, 46, 50]. Overall, these studies supported the existence of site-specific features in digestive NEC, which show similarities with non-neuroendocrine carcinomas of the same locations [35, 46, 50]. Interestingly, is has been suggested that large cell NEC were genetically more similar to adenocarcinomas arising in the same site, compared to small cell NEC [46], highlighting the utility of the morphological distinction of the two subtypes endorsed by the last WHO classification of NEN [8]. In the study by Venizelos et al. [46], *TP53*, *APC*, *KRAS*, *BRAF*, and *RB1*, in order of frequency, were confirmed to be the most mutated genes across digestive NEC, with few other genes involved (*KMT2D*, *FBXW7*, *GNAS*, *ARID1A*, *NF1*, and *CTNNB1*). In turn, Yachida et al. reported *TP53*, *KRAS*, *RB1*, *CCNE1*, *CDKN2A*, *APC*, and *MYC* as significantly altered genes in digestive NEC [35]. Interestingly, it was highlighted that NEC with Ki67 PI lower than 55% harbored an enrichment of MYC CNA [46]. Moreover, two thirds of the whole series of digestive NEC exhibited one or more druggable molecular alterations [46]. Going into depth of the differences among NEC of different molecular sites, pancreatic NEC showed higher numbers of structural variations, as well as of non-synonymous mutations [35]. Importantly, alterations of the *Notch* family genes were found exclusively in non-pancreatic NEC [35], whereas *BRAF* mutations were more frequently found in colonic NEC than other locations [46, 64], alterations of the WNT/beta-catenin pathways were found in more than 50% of gastric NEC [50], and *RB1* alterations were more common in the pancreatic site, with lower frequencies in colonic, rectal, and gastric NEC [46], as it was already reported by others [56, 58]. In non-pancreatic NEC with no alterations in *RB1*, *MYC*, and *CCNE1* were mutually exclusive events [35]. When MSI was investigated, a small subset (about 5%) of NEC resulted instable, with a slight prevalence of colorectal NEC, where MSI was significantly co-occurring with *BRAF* mutation [46, 64]. Focusing on pancreatic NEC, Yachida et al., reported the existence of two different clusters, “ductal-type” and “acinar-type” based on a multiomic approach [35]. The ductal-type NEC consistently showed *RB1* and *TP53* alterations and shared *KRAS* mutations and ductal marker expression (*SOX2*, *ASCL1*, *NKX2.1*, *EZH2*, and *E2F1*) with ductal pancreatic adenocarcinoma, but lacked alterations of p16 and SMAD4 expression. This suggests divergent pathogenetic pathways for the neuroendocrine and non-neuroendocrine counterparts [35]. In turn, the acinar-type NEC showed absence of *KRAS* mutations, alterations of *APC*, *CCND1*, and *CDK2NA* genes and overexpression of PTF1A, GATA4, NR5A2, and RBPJL transcription factors [35]. Importantly, pancreatic NEC lacking *KRAS* mutation had a significantly better survival than those with *KRAS* alteration [35]. Figure 1 summarizes site-specific alterations in digestive NEC.

### Common Molecular Alterations in Pulmonary and Digestive NEC: Bases for a Molecular Classification?

Digestive and pulmonary NEC show a substantial morphological overlap that is mirrored by common molecular alterations. The high frequency of *TP53* and *RB* alterations with loss of function of this important tumor suppressor genes is considered crucial for the shift towards a neuroendocrine phenotype in the pathogenesis of both groups of neoplasms [65]. Importantly, co-existence of *TP53* and *RB* loss of function is a feature of NEC that is not shared with non-neuroendocrine carcinoma of the same sites [60]. Although *RB* mutation is consistently less frequent in digestive NEC than in their pulmonary counterpart [10, 35, 46], other genomic or non-genomic mechanisms have been shown to be responsible for the inactivation of the Rb pathway, confirming its pivotal role in these neoplasms [60, 66].

Recently, a molecular classification of pulmonary NEC has been proposed, according to the differential expression of transcriptional drivers that regulate neuroendocrine phenotype. Small cell NEC of the lung have been subtyped in four subgroups, identified by the preferential expression of either *ASCL1*, or *NEUROD1*, or *POU2F3*, or *YAP1* genes [67]. Similarly, large cell pulmonary NEC have been classified into two groups, namely *ASCL1*/*DLL3*-high and *NOTCH1*-low and the *ASCL1*/*DLL3*-low and *NOTCH1*-high [68]. This molecular classification is related to diverse expression of neuroendocrine phenotype in the various subtypes and underlies the pathogenetic activation of distinct intracellular pathways [67, 68]. Such new knowledge paves the way for the clinical employment of targeted drugs interacting with molecular pathways related to the specific transcription factors activated in each NEC class, skipping the dogma of lung NEC as a monolithic disease, with aggressive platinum-based schedules as the only available therapy [69, 70]. Moreover, the use of immunohistochemistry as surrogate of molecular analysis for the study of these transcription factor is available and affordable, making the molecular classification of lung NEC feasible in every Pathology department [71]. In an experimental setting, an elegant study establishing a biobank of patient-derived organoid of digestive NEN showed clustering according to the expression of ASCL1/NEUROD1, NKX2-5, and POU2F3, evoking the subtypes described by Rudin on the lung, but with differences related to the preferential NKX2-5 expression in digestive NEC, compared to ASCL1 and POU2F3, which are frequently expressed in lung NEC [49]. Importantly, a recent work by the Turin group was able to confirm, in a clinical series, a certain overlap between the transcriptional profile of pulmonary and extrapulmonary NEC, including digestive NEC and NEC of other sites [51]. Besides the important potential therapeutic correlates, as discussed before, this study highlighted two other important points. First, it supports the vision that a “neuroendocrine-specific phenotype” is present across NEC of different anatomical sites; second, it suggested that the differential activation of transcription factors related to the modulation of neuroendocrine phenotype may also be related with prognosis [51]. These concepts were further supported by a Korean study, in which YAP1-expressing and PUO2F3-expressing extra-pulmonary NEC were further characterized in terms of possible molecular target therapy [52].

## Molecular Features of Digestive NET

**Fig. 2:**
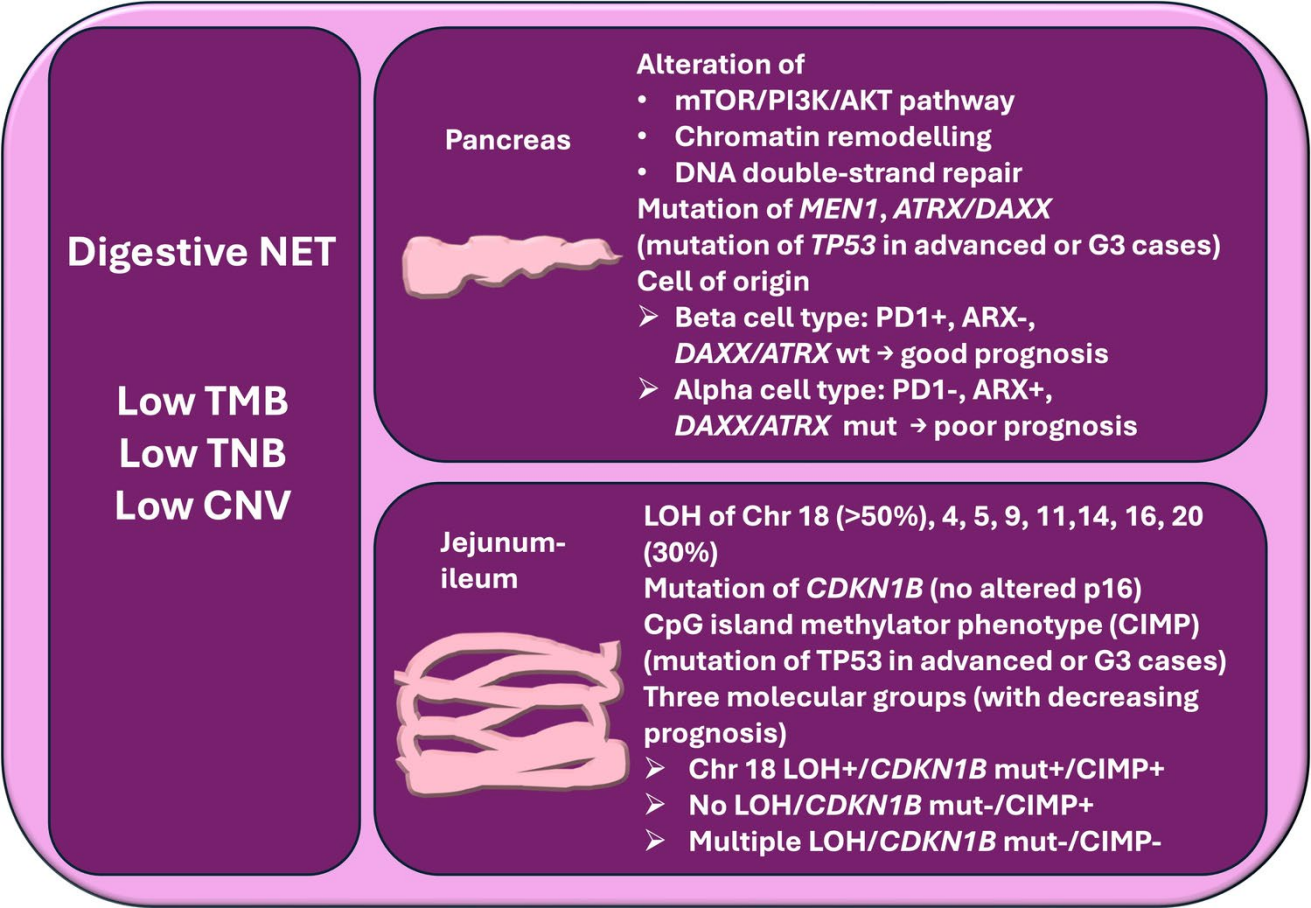
Summary of site-specific molecular alterations in digestive NET. TMB, tumor mutation burden; TNB, tumor neoantigen burden; CNV, copy number variation; wt, wild type; mut, mutated; LOH, loss of heterozygosity

Most of the molecular studies available on digestive NET have been performed on pancreatic NET (PanNET), from which we have learned important general lessons on NEN, that are guiding actual therapeutic approaches with biological drugs [72]. The introduction, in the clinical practice, of drugs targeting, for example, somatostatin receptors (both in the form of medical and peptide related radio-nuclide therapy), mTOR/PI3K/AKT pathway, MGMT, and hypoxia-related mechanisms have reoutlined the approach to advanced NET and have given new chances to the patients, and also the impact on diagnostic procedures (functional imaging) cannot be underestimated [73, 74]. However, the molecular landscapes of PanNET and non-pancreatic digestive NET, for different reasons, need to be further explored. In PanNET, the great morphological, functional, and prognostic variabilities of these neoplasms have raised the need of better focusing on pathogenetic mechanisms in order to identify possible molecular targets for even more tailored treatment. Molecular knowledge about non-pancreatic digestive NET is not as established as for PanNET, and the “pancreatic molecular paradigm” seems to fit less well for these tumors [75]. As for intestinal NET, one should first recognize that this group of neoplasms is composed by distinct entities, identified by anatomical sites. Broadly, we can affirm that duodenal NET, jejunum-ileal NET (also including rare right colon NET), appendicular NET, and rectal NET represent different nosological entities [20]. NET of the jejunum-ileum is, together with PanNET, the most common NEN in the digestive tract [20], and molecular studies have been performed on large case series, highlighting distinct profiles compared to PanNET [75]. Regarding digestive NET of the stomach, the duodenum, the appendix, and the colon-rectum, systematic molecular studies analyzing the genomic landscape of these neoplasms are lacking, due in part to their rarity and in part to the generally indolent behavior of many of them, which does not usually require medical therapy for advanced disease. Thus, only few data are available, suggesting, in small subsets of these tumors, the presence of gene alterations that might have a prognostic or predictive value, generally related to and increased proliferation index [76, 77]. In the following text, the available knowledge about molecular profiles of pancreatic and jejunum-ileal NET will be summarized (Fig. 2).

### Molecular Alterations of PanNET: Clustering Towards a Molecular Classification

The multifaceted panorama of PanNET in the context of NET may only be mirrored by pituitary NET, in terms of the number of entities that may be distinguished based on morphology, endocrine functionality, and oncological aggressivity [4, 9]. Thus, a similar heterogeneity is conceivable for the molecular alterations that are enfolded in the warp and weft of each of these entities. Here, we will not address the molecular features of PanNET occurring in the context of hereditary cancer syndromes, whereas we will concentrate on the latest acquisitions about sporadic PanNET. However, as already mentioned in this text, it is worth remembering that genes involved in hereditary PanNEN, including but not limited to *MEN1*, *TSC1* and *2*, and VHL, may be found altered or mutated in sporadic PanNEN with morphological, prognostic, and predictive correlates [14, 78, 79]

A milestone in the understanding of heterogeneity in PanNET was recently settled by analyzing the prognostic value of *MEN1*, *ARTX*, and *DAXX* gene mutations in non-functioning PanNET and the gene expression profile of mutated and non-mutated cases for these genes. It was demonstrated that the presence of mutation of *MEN1*, *ARTX*, or *DAXX* genes was related to a worse prognosis than in wild-type cases [80–83]. Moreover, that the gene expression profile of tumors with *MEN1*, *ARTX*, and *DAXX* gene mutations was shown to be similar to that of alpha cells of pancreatic islets [80]. In turn, non-functional (NF) PanNET with wild type *MEN1*, *ARTX*, and *DAXX* genes showed a gene expression profile that was variably similar to that of other islet cell types (beta, PP, delta, etc.), thus establishing the concept of a putative cell of origin for these neoplasms [80]. The possibility of distinguishing at least two groups of PanNET according to the alpha or beta cell of origin and the prognostic value of this distinction have been also confirmed by the analysis of methylome in a large series of cases [84]. Of practical importance, the alpha-cell profile showed by *MEN1*, *ARTX*, and *DAXX* mutant cases included the expression of *ARX* and *HNF1A* genes, which are involved in pancreatic alpha cell function, whereas they lacked the expression of PDX1, which is typically expressed in beta-cells and transcriptionally repressed through promoter hypermethylation in alpha cells [80]. The prognostic value of the differential expression of ARX and PD1 was also demonstrated in a cohort of 103 NF PanNET, in which almost all metastatic cases were ARX + PD1- (alpha-cell type), all of them with alternative lengthening of telomerase alterations (ALT), due to the alternative loss of *DAXX* or *ATRX* genes [85]. In contrast, PD1 + ARX- cases (beta-cell type) showed poor propensity to metastatic dissemination and a longer overall survival [85]. Nevertheless, a recent study, analyzing a large international cohort of more than 1000 pancreatic and extra-pancreatic NET, besides assessing the specificity of *DAXX/ATRX* alterations for the pancreatic site, supported the independent prognostic role of *DAXX/ATRX* alterations, but not that of PDX1/ARX [86].

The good prognostic meaning of a beta-cell gene expression profile seems to be confirmed by the indolent behavior of insulinoma, regarding its metastatic potential and the post-surgical overall prognosis. Indeed, several studies demonstrated that insulinoma and NF PanNET harbor distinct recurrent gene mutation. Namely, mutations of *MEN1*, *DAXX/ATRX*, and mTOR pathway genes, frequently found in NF PanNET, were seldom detected in insulinoma, in which mutations of *YY1* gene, not involved in NF PanNET, were found in a non-negligible fraction of cases [14, 87–90]. The following studies confirmed the genetic differences between insulinomas and NF PanNET, highlighting other, more general, genomic differences between the two, which are related to copy number variations (CNV) and epigenetic profiles [91]. Interestingly, Hong et al. showed that *YY1* mutated insulinomas harbored neutral CNV, those with *YY1* wild type tended to have CNV amplification, with frequent involvement of chromosome 7, whereas no case of CNV deletion was found among insulinomas [91]. Regarding NF PanNET, those with CNV amplification/deletion were likely to have a worse prognosis, particularly if *DAXX/ATRX* mutations were present [91]. The majority of insulinomas do not represent an oncological problem, as they do not locally progress or metastasize, the need for surgery being based on the exigence of controlling the endocrine hyperfunction. Nevertheless, a small proportion of insulinomas may behave aggressively, presenting with large masses and metastatic disease. Hackeng et al. explored the expression profiles of aggressive versus indolent insulinomas and demonstrated that, also in these NET, expression of ARX and presence of ALT, in absence of PDX1 expression is related with higher rates of recurrences and metastatic dissemination [92].

In summary, the study of molecular alterations in PanNET paints a complex picture in which the interactions between genetic and epigenetic abnormalities intertwine and appear, to some extent, to be interdependent. In this context, it is also worth recalling the event of inactivation of O6-methylguanine-methyltransferase (MGMT) via promoter methylation, which has been reported to be more frequent in grade 2 PanNET and predicts a good response to alkylating antineoplastic drugs [93, 94].

### Molecular Alterations of NET of the Jejunum/Ileum (JINET): A Play Yet to Be Written?

NEN arising in the jejunum/ileum are a unique group of neoplasms among digestive tract NEN, under several point of views. First, JINEC are only anecdotally reported [95, 96], and NET are virtually the only type of NEN in this anatomical site [20]. Second, JINET are almost exclusively represented by serotonin-producing EC cell tumors, with virtually no other NET type described in this site, except for exceedingly rare cases of gastrin-producing G cell tumors (gastrinomas) in the upper jejunum [97]. Third, compared to EC cell tumors of other digestive sites, i.e., the appendix and the rectum, EC cell JINET show distinctive features namely the worse prognosis related to higher propensity to deep infiltration of the intestinal wall and to metastatic dissemination [98–102], and the frequent association with fibrotic changes, including, but not limited to, mesenteric and cardiac fibrosis [103]. These peculiar features, and the reasons for their site-specificity in presence of overlapping morphological appearance of EC cell NET in different anatomical sites, still remain substantially unexplained. Regarding fibrosis, microenvironment composition, growth factors activity, and intracellular signaling pathways have been explored the pathogenesis as possibly involved in [103, 104]. Fourth, although they frequently present with advanced and metastatic disease, most of JINET are grade 1 tumors, with Ki67 PI (PI) lower than 3%. In fact, Ki67 PI has not proven to be able to predict metastatic potential of JINET, albeit increasing values of this index have been reported to be related with and increased risk of disease progression and death for disease [21, 105]. Fifth, up to 50% of JINET presents with multifocal disease with independent clonal origin [106], without the presence of a known genetic cancer predisposition syndromes, although a familial predisposition has been hypothesized, based on the finding, in these tumors, of rare gene alterations related to hereditary syndromes [107–109]. Moreover, it is worth noting that JINET arise in an intestinal tract that is very rarely involved by primary malignancies, hosting less than 2% of all digestive cancers [110], suggesting a particular setting in relation to risk factors.

In such a complex and remarkable scenario, the genetic landscape of JINET is surprisingly deserted, and significant research reports about this topic are very few in the literature, compared to those regarding PanNET [111]. JINET have been reported to have the lowest tumor mutation burden (TMB) among adult human malignancies, with virtually absent recurrent gene mutations [112]. In 2013, the finding in a subset of JINET of mutated *CDKN1B*, encoding for p27 protein and related to MEN4 syndrome, was hailed as the beginning of a new molecular era in the management of these tumors [113]. Although it soon became evident that mutation of this gene was present in less than 10% of cases of JINET [114–116], data on clinical and experimental samples showed that the loss of one allele was enough to drive JINET pathogenesis [117]; thus, it was suggested that nearly 20% of cases might be driven by the alteration of *CDKN1B* [118]. Subsequent studies, however, demonstrated that this gene was more frequently mutated in advanced disease, occurring in later stages of the natural history, and possibly not being associated with an early tumorigenic activity or aggressivity [32, 119]. Other, even less frequent, non-recurrent mutations in JINET are *APC*, *CKDN2C*, *BRAF*, *KRAS*, *PIK3CA*, *TP53*, and other oncogenes and tumor suppressor genes that, however, have been mostly detected in widely metastatic tumors and seem to be associated with increased proliferation index in progressive disease [32, 120]. It has been proposed that these genetic alterations may still be considered as molecular targets for therapy of advanced JINET [120]. However, it should be borne in mind that these infrequent gene mutations in JINET may be the expression of the high intratumor heterogeneity of these neoplasms, that has been reported both in primary and in metastatic lesions [121] and they may not represent truly meaningful biomarkers for effective patient management.

In contrast with the low numbers of genetic alterations, JINET frequently show recurrent chromosomal abnormalities, involving whole chromosome or chromosome arms. More than half of JINET show loss of chromosome 18 [122, 123]. Notwithstanding this consolidated piece of data, important tumor-related genes located on this chromosome, including but not limited to *BCL2*, *DCC*, *CDH19*, and *SMAD4*, have not been found to be involved in the pathogenesis of JINET, and chromosome 18 loss remains still to be interpreted in a mechanistic perspective [116, 124]. Recently, it has been suggested that the presence or absence of chromosome 18 loss in JINET may be associated with differential expression of genes like *AMPD3* and *KCNMB2* and to a different composition of the tumor microenvironment [125]. Additional recurrent chromosomal abnormalities in JINET involve gain of chromosomes 4, 5, 14, and 20 and losses of chromosomes 9, 11, and 16 [126, 127], but no candidate gene located in these chromosomes has proven to drive JINET pathogenesis.

Despite the paucity of genetic alterations, epigenetic changes and expression profiles have been reported to be frequent in JINET. In general, CpG island methylator phenotype (CIMP) has been observed in more than half JINET [128], whereas differential promoter methylation of certain genes, i.e., *CTNNB1* and *RASSF1A*, has been shown to be involved in progression and metastatic dissemination of these tumors [129]. Importantly, different tumor methylation profiles are able to stratify a patient’s risk [128, 130, 131]. Similarly, transcriptomic profiles have been reported to identify distinct subgroups characterized by different biology and potential therapeutic targets [132]. In addition, gene expression profiling, supported by functional analysis in cell lines and animal models, has identified *EZH2* as a candidate oncogene in JINET, with important potential implications for therapy, as *EZH2* is a target for metformin [133].

Although no single genomic alteration has been reported to have a significant prognostic or theragnostic value in JINET, the integration of large-scale chromosomal abnormalities (copy number alterations, CNA), *CDKN1B* mutations, and CIMP phenotype has proven to be effective in identifying three groups of tumors with different prognosis [131]. Specifically, group A, with good overall prognosis, was represented by JINET with loss of chromosome 18 and *CDKN1B* mutation but did not show CIMP phenotype. Group B, with intermediate prognosis, included JINET with no CNA, no *CDKN1B* mutation, and CIMP phenotype. Finally, group C featured multiple CNA and was characterized by poor prognosis [131].

## From the Bench to the Bedside: Clinical Application of Molecular Knowledge in Digestive NEN—Are We Ready for a Molecular Classification of Digestive NEN?

The medical treatment of digestive NEN is reserved to advanced, inoperable or metastatic, disease and is aimed to prolong patients’ survival. Although it may seem obvious, the most important parameter on which therapy is currently based is the distinction between NEC and NET. In fact, the former are treated with platinum-based chemotherapy combined with etoposide, inspired by the similarity to their pulmonary counterpart [134, 135], whereas NET are basically resistant to chemotherapy schedules used for epithelial malignancies commonly seen in the digestive tract (i.e., adenocarcinoma) [72]. This different response to traditional anti-neoplastic drugs is easily understandable in the light of the very high proliferation rate of NEC, compared to the distinctively low proliferation rate of NET. However, this is everyday experience of clinical practice; NEC invariably recur in a short time despite the initial response to chemotherapy and eventually kill the patient [134]. On the other hand, despite the well differentiated morphology, the low proliferation index, and the claimed “indolent” behavior of NET, a significant proportion of patients experience advanced disease at diagnosis or metachronous metastases and need to be treated with medical therapy [72]. From here, the need to find new and effective therapeutic strategies arises, and the molecular-based therapeutic approach opens new perspectives in this sense. The systematic discussion of the molecular targeted therapeutic strategies employed for NEN is beyond the scope of this article and may be found in several comprehensive reviews by other authors [see refs 73, 137–139 as non-exhaustive examples]. In this paragraph, however, I will review molecular alterations representing targets for clinically employable drugs and, thus, individuating classes of neoplasms amenable to different specific therapies.

In the light of recent acquisition about the molecular landscape of digestive NEC, alternative therapeutic strategies to classical chemotherapy and several “druggable” targets have emerged.

- The existence of site-specific differences among digestive NEC and the similarities with non-neuroendocrine carcinomas of the same anatomical locations has prompted the use of “adenocarcinoma-like” chemotherapy schedules. Ongoing trials are multiplying, with initial promising response and acceptable toxicity [136–138]. In addition, the detection of altered genes and cellular pathways (e.g., *BRAF* mutation and MSI in colorectal NEC, *RAS* mutations in pancreatic NEC, and *MYC* amplification in gastric NEC) may represent the rationale for the employment of specifically targeted drugs [136, 139]
- The virtually ubiquitous alterations of *TP53* and *RB* genes, whether due to somatic mutations or to other inactivating mechanisms, cause a consistent dysregulation of cell cycle in NEC and DNA damage repair mechanisms (DDR). This situation is related to sensitivity to platinum-etoposide-based therapy and may be worsened by somatic mutations or other alterations of genes involved in these important cellular functions, such as Aurora kinase, CHK1, and PARP proteins. Drugs targeting DDR components have been already used for treating other cancer types in combination with chemotherapy and have been tested in pulmonary and prostatic NEC, representing a theorical option also for digestive NEC [140–142].
- The lung NEC-like molecular classification of digestive NEC, based on the differential expression of *ASCL1*, *NEUROD1*, *POU2F3*, and *YAP1* genes, paves the way for the use of specific drugs targeted against molecular components of the cellular pathways regulated by these transcription factors. *ASCL1*-driven NEC may be sensitive to inhibitors of BCL2 and DLL3 [143, 144]; *NEUROD1*-driven NEC have been showed to be sensitive to Aurora kinase inhibitors and drugs targeting PI3K/mTOR pathway [145–147]; *POU2F3*-driven NEC seem to be sensitive to IGF1R inhibitors, albeit these drugs have only been experimented preclinically [69]; finally, in *YAP1*-driven NEC that are considered chemotherapy-resistant, PARP inhibitors have been tested in combination with chemotherapy with no definite result [69, 148]. It is worth noting that these data are obtained in clinical trials involving only pulmonary NEC and only preclinical results are available on digestive NEC [49, 149].
- NEC are potentially immunogenic neoplasms, in relation to their high tumor mutation burden, the DNA damage repair defects, and, in a subset of cases, to microsatellite instability. Based on this rationale, immunotherapy with immune check point inhibitors (ICI) has been employed in patients with NEC of the lung and is currently being tested in extra-pulmonary NEC, including digestive ones. Clinical trials with anti-PD1, anti PDL-1, and anti-CTLA4 drugs, in monotherapy or in combination, are ongoing with conflicting results [150], and the employment of these therapeutics should be judiciously evaluated since they may give serious adverse effects on vital organs and systems [151]. Importantly, immunogenicity of NEC may be increased or elicited using DNA-damaging drugs, such as antiblastic chemotherapeutics, and the association of ICI with standard chemotherapy has given good results in terms of overall survival [136, 152, 153].

In fact, molecular-targeted therapy for NET has a more consolidate history than for NEC and has been employed since the approval of somatostatin analogs for treatment of metastatic disease in 2009 [154, 155]. Soon thereafter, the tyrosine kinase inhibitor sunitinib with anti-VEGF activity was tested and approved, based on the morphological evidence of the rich vascularization of NET and in the attempt to inhibit neoangiogenesis [156]. Since these initial empiric approaches to precision medicine in NET, the ever-growing molecular insight of the last two decades, sustained by the high throughput technologies for genomic analyses, has laid down the rationale for the employment of new generation drugs, targeting specific genes, pathways or mechanisms involved in the pathogenesis of these tumors. As already mentioned, the discussion of specific drugs and their clinical setting of application is not the aim of this review, and the reader is referred to extensive addressing of this topic elsewhere [72, 73, 157]. In a nutshell, the list of targetable mechanisms is reported here:

- PI3K/AKT/mTOR pathway (targeted by molecules such as Everolimus, Dactolisib, Alpelisib, and analogs)
- DNA double strand break repair (targeted by PARP inhibitors, such as Olaparib)
- DNA single strand break repair (driven by MSI and *MUTYH* gene alterations, may be amenable to immunotherapy)
- Chromatin remodeling and alternative telomere lengthening, ALT (targeted, among others, by histone deacetylase inhibitors as Panabinostat, DNA methyltransferase as ASTX727, and ARID1A inhibitors as Tazemezostat)
- Cell cycle modulating pathways (targeted by inhibitors of CDX4/6, DNA protein kinase, and serine/threonine protein kinases)
- Angiogenesis and hypoxia-related pathways (targeted by inhibitors and specific antibodies directed against of a variety of growth factor receptors and other tyrosine kinase, including but not limited to VEGFRs, PDGFRs, KIT, MET, and RET, as well as hypoxia inducible factors, HIFs)
- WNT/beta-catenin pathway (experimentally targeted in preclinical studies by antibodies against FZD receptors, porcupine inhibitors, tankyrase inhibitors, and Dvl inhibitors)

An important challenge in the therapy of NET is the management of grade 3 neoplasms that have been demonstrated to have some degree of biological and clinical overlap with NEC [45] and must be correctly diagnosed and distinguished from both NEC and G2 NET [8]. G3 NET are not effectively treated with currently available “biological drugs” and need to be treated with chemotherapy, in the setting of advanced disease. They show good response to capecitabine/temozolomide combinations but are still amenable to chemotherapy with platinum and etoposide [30, 158]. It is worth noting that temozolomide is an alkylating agent, the action of which is party antagonized by MGMT. Thus, the inactivation of MGMT by promoter methylation is associated with a good response to this drug, providing the rationale for investigating protein expression at the tissue level [93, 94].

Very recently, preclinical models for testing of gene mutation-specific drugs in NEN have been established, including cell lines and organoids, further expanding the possibilities for a really personalized treatment of these neoplasms [49, 149, 159]. However, at this point it should be recognized that, notwithstanding the plethora of information about the genomic landscape of NEN, the gathered data represent a good foundation but are still not definitely sufficient for a robust molecular classification of digestive NEN. While molecular studies have confirmed, supported, and informed the pathological classification of NEN, clarifying the differences between NET and NEC and helping to define the G3 NET category, they do not seem to identify criteria for a “stand-alone” molecular definition of nosological entities, as seen in other types of tumors (e.g., breast cancer, gastric cancer, colorectal cancer, endometrial cancer, and others). Consequently, a true molecular-based precision therapy approach is not yet feasible for these neoplasms. Although genetic drivers of NET and NEC have been partially identified, the application of truly targeted therapy based on molecular data remains an objective rather than an established fact. This is surely due to the rarity of these neoplasms, which hampers the conducting of large-scale randomized trials with specific drugs that would integrate molecular studies into real-life clinical settings. Additionally, it is becoming increasingly clear that molecular testing should be performed using genome-wide analyses rather than focusing solely on known specific alterations. However, this approach is still highly expensive in the face of limited funding for such rare neoplasms. Moreover, the frequent changes in the nomenclature and classification of NEN have not facilitated the collection of uniformly diagnosed cases and may have acted as a confounding factor. Finally, there is also a scarcity of preclinical studies on animal models, cell lines, and patient-derived organoids for the identification of molecular targets and drug testing. Large-scale multicenter studies are needed, based on pathologically well-characterized case series, analyzed with comprehensive genomic technologies, and supported by preclinical drug screening and clinical trials. Only through this approach will the molecular classification of digestive NEN become an independent reality rather than merely a reflection of clinical and morphological features.

## Data Availability

No datasets were generated or analyzed during the current study.

## Declarations

### Peer Review Information

This paper has been peer-reviewed as per the journal's standards.
